# Functional recovery of odor representations in regenerated sensory inputs to the olfactory bulb

**DOI:** 10.3389/fncir.2013.00207

**Published:** 2014-01-07

**Authors:** Man C. Cheung, Woochan Jang, James E. Schwob, Matt Wachowiak

**Affiliations:** ^1^Department of Biology, Boston UniversityBoston, MA, USA; ^2^Department of Anatomy and Cellular Biology, Tufts University School of MedicineBoston, MA, USA; ^3^Brain Institute and Department of Neurobiology and Anatomy, University of UtahSalt Lake City, UT, USA

**Keywords:** olfactory bulb, regeneration, sensory neurons, synaptopHluorin, axon targeting

## Abstract

The olfactory system has a unique capacity for recovery from peripheral damage. After injury to the olfactory epithelium (OE), olfactory sensory neurons (OSNs) regenerate and re-converge on target glomeruli of the olfactory bulb (OB). Thus far, this process has been described anatomically for only a few defined populations of OSNs. Here we characterize this regeneration at a functional level by assessing how odor representations carried by OSN inputs to the OB recover after massive loss and regeneration of the sensory neuron population. We used chronic imaging of mice expressing synaptopHluorin in OSNs to monitor odor representations in the dorsal OB before lesion by the olfactotoxin methyl bromide and after a 12 week recovery period. Methyl bromide eliminated functional inputs to the OB, and these inputs recovered to near-normal levels of response magnitude within 12 weeks. We also found that the functional topography of odor representations recovered after lesion, with odorants evoking OSN input to glomerular foci within the same functional domains as before lesion. At a finer spatial scale, however, we found evidence for mistargeting of regenerated OSN axons onto OB targets, with odorants evoking synaptopHluorin signals in small foci that did not conform to a typical glomerular structure but whose distribution was nonetheless odorant-specific. These results indicate that OSNs have a robust ability to reestablish functional inputs to the OB and that the mechanisms underlying the topography of bulbar reinnervation during development persist in the adult and allow primary sensory representations to be largely restored after massive sensory neuron loss.

## Introduction

The mammalian olfactory system has a remarkable capacity for regeneration of its primary sensory neurons (olfactory sensory neurons; OSNs) after loss due to injury, infection, or exposure to toxins. Even after a virtually complete loss of all OSNs, the population is restored to a level nearly indistinguishable from the original in terms of neuronal number and topography of odorant receptor (OR) protein expression (Schwob et al., 1999; Iwema et al., 2004). These newly-generated OSNs must reestablish convergent axonal connections with their appropriate targets in the olfactory bulb (OB). During normal development, the axons of all of the several thousand OSNs expressing the same OR converge onto just a few (2–4) of the ~1600 glomeruli in the OB (Mombaerts et al., 1996). Because individual glomeruli serve as functional units in the central coding and processing of odor information, reestablishing appropriate connections between OSNs and glomeruli is likely critical for normal olfactory function. For example, errors in the reinnervation of glomeruli may underlie olfactory dysfunction in humans recovering from olfactory loss due to trauma or infection (Doty, 1979; Meisami et al., 1998). More generally, reestablishing appropriate neural connectivity is a prerequisite for the full recovery of function of any sensory or motor system.

Previous studies have demonstrated that, in the adult, the targeting of OSN axons to glomeruli after lesion is subject to errors that do not occur during development (Schwob et al., 1999; Costanzo, 2000; St. John and Key, 2003; McMillan Carr et al., 2004; Blanco-Hernández et al., 2012). These errors include a lack of exclusive convergence of OSNs onto the same glomerulus and projection of at least some axons of a given OSN population to multiple, dispersed glomeruli (Costanzo, 2000; St. John and Key, 2003; Blanco-Hernández et al., 2012). The impact of this mistargeting on odor representations remains largely unclear, however: targeting has been examined for only three OR-defined group of OSNs out of the approximately 1000 ORs expressed in the rodent olfactory system (Gogos et al., 2000; St. John and Key, 2003; Blanco-Hernández et al., 2012). Thus there is no generalized picture of the effect of OSN loss and regeneration on functional odor representations in the CNS.

To address this question, we used mice expressing synaptopHluorin in OSNs (Bozza et al., 2004) to functionally assess how odor representations recover after lesioning the olfactory epithelium (OE) with the olfactotoxin methyl bromide (Schwob et al., 1999). We found that despite apparent errors in the exclusive convergence of OSNs onto glomeruli, odor representations involving multiple glomeruli largely recovered after lesion, with a topographic organization and overall magnitude similar to that seen before lesion. These results indicate that the olfactory system shows a robust capacity to regenerate functional inputs to the CNS in a manner that, in all but the most severe cases, preserves the broad spatial organization of odor representations that was present before injury. Thus, primary representations of odor information can be largely reconstituted in the adult even after large-scale neuronal loss, an ability unique among sensory systems.

## Materials and Methods

### Animal strains and care

We used olfactory marker protein-synaptopHluorin (OMP-spH) mice (Bozza et al., 2004) that had been backcrossed into the C57/Bl6 strain and bred with the 129/SvImJ strain to generate first generation (F1) hemizygous mice. Only males were used in the study due to their sensitivity to methyl bromide. The F1 animals were housed under standard conditions in ventilated racks until 12 weeks of age before being exposed to MeBr. All mouse colonies were bred and maintained at the Boston University animal care facility. Animals were transported to Tufts University School of Medicine for exposure and were returned to Boston University on the same day. All surgical and experimental procedures were approved by the Boston University and Tufts University Institutional Animal Care and Use Committees.

### Surgery

Seven to ten days before lesioning, custom made head caps were implanted on the skull using aseptic surgical procedures. The head cap consisted of a custom-milled aluminum plate that fit the skull snugly and to which two M2 bolts were attached. Animals were anesthetized with isoflurane, placed in a stereotaxic holder and the head cap was placed with its anterior edge aligned to the coronal suture and attached to the skull using dental acrylic. A piece of 30 gauge metal tubing was embedded in the dental acrylic posterior to the OB to serve as a fiducial marker for chronic imaging experiments. The acrylic, which was darkened to reduce autofluorescence, was extended from the head cap to the frontal-nasal fissure, forming a well surrounding the dorsal OB.

For 3 days after surgery, wound margins were treated topically with the anesthetic bupivacaine (1 mg/ml, Sigma-Aldrich, St. Louis, MO) and cleaned with Betadine. Animals were also injected with the nonsteroidal anti-inflammatory carprofen (5 mg/kg SC, Pfizer, New York, NY) and the antibiotic Baytril (3 mg/kg, IM). Animals were monitored closely for a 2 week span following surgery, the first imaging session, and MeBr exposure. Animals were given carprofen and Baytril as necessary.

### Chronic and acute optical imaging

Animals outfitted with head caps were imaged at a minimum of three time points: a “pre-exposure” session to obtain baseline odor response maps, a “post-exposure” session 4 days after exposure to determine whether MeBr successfully induced lesions, and a final “recovery” session 12–13 weeks after exposure to determine the extent of regeneration and recovery of functional responses.

Immediately prior to pre-exposure imaging (minimum 3 days before MeBr exposure), animals were anesthetized with isoflurane and placed in a custom head holder mounted on goniometers and *x*- and *y*-translation stages that allowed for independent positioning and rotation of the animal. The bone over the OBs was thinned to transparency, sparing a thin wall of dental acrylic surrounding the OB to form a well around the imaging window. After the first imaging session, the bone was dried and a layer of ethyl 2-cyanoacrylate glue (Instant Krazy Glue, KG925, Elmer’s Products, Inc., Columbus, OH) was applied to the window. After the glue had set, the well was filled with silicone sealant (Kwik-Sil or Kwik-Cast, World Precision Instruments, Sarasota, FL). In nearly all cases, the combination of ethyl cyanoacrylate and silicone based adhesive preserved window translucency for approximately 1 month. There was more variability in preservation of window quality for the ~14 week period of the study. When windows were no longer translucent at the recovery time-point, the bone was re-thinned prior to imaging.

OB alignment across repeated imaging sessions was performed using one of two systems. For the first system, the platform position was fixed relative to a custom objective mount using an alignment bar. The micrometer translation stages allowed for adjustments relative to the initial fixation point. For the second method, a fiducial marker was embedded in the dental cement of the head cap. During the first imaging session, an image of the fiducial marker was taken for later alignment. The marker was placed so that it was visible in the field of view when the OB was centered under the objective.

For acute imaging sessions, anesthetized animals were placed on the custom head holder and the bone over the OB was thinned to transparency and a coverslip and mouse Ringer’s solution placed over the OBs.

### Olfactometry

Odorants were selected and delivered using a 16 channel computer-controlled olfactometer, as described previously (McGann et al., 2005). Odorant concentration across imaging sessions was confirmed before each session using a portable photoionization detector (miniRAE 2000, RAE Systems, San Jose, CA). All olfactometer parts (including the odorant chambers and anesthesia mask) were made from Teflon or PTFE tubing. Isoflurane was used as an anesthetic to maximize survival across multiple imaging sessions. Isoflurane and odorant were delivered to the animal through a custom anesthesia/odorant delivery mask that fit tightly around the mouse’s snout. Isoflurane was vaporized (EZ-155, Euthanex Corp, Palmer, PA) and mixed with medical grade oxygen. To maintain constant oxygenation levels throughout the experiment, a solenoid was used to pass odorized air into the gas mask during odor presentation and filtered air between trials. The filtered air was set to match the flow rate of the odor line. Thus, the total flow was maintained at 0.9 L/min during and between odor presentation (0.5 L/min isoflurane and oxygen with either 0.4 L/min air or odorized air). In acute experiments, we used a conventional concentric delivery nozzle, described previously (Lam et al., 2000). In this case, total air flow was 0.5 L/min.

Odorants used (and their purities) included 2-hexanone (98%), 2-butanone (99.5%), ethyl butyrate (99%), methyl valerate (99%), *trans*-2-methyl-2-butenal (96%), isovaleraldehyde (97%), 2-aminoacetophenone (98%), hexyl acetate (99%), benzaldehyde (99%), phenylacetaldehyde (90%), and methyl salicylate (99%) from Sigma-Aldrich; butyric acid (99.5%) and butyl acetate (99.5%) from MP Biomedicals Inc.; and eugenol (99%), menthone (97%), acetophenone (98%) and methyl benzoate (98%) from Fluka.

### Methyl bromide lesion

Animals were exposed to MeBr as previously described (Schwob et al., 1995, 1999; Chen et al., 2004). F1 OMP-spH heterozygous (C57/Bl6 × 129SvImJ) males were exposed unilaterally to MeBr at 12 weeks of age. One side was protected by insertion of a plug made from polyethylene tubing and suture (Cummings et al., 1997) and sealed at the external naris with superglue. Animals were placed into a Plexiglas chamber and exposed to MeBr gas (Matheson Gas Products, East Rutherford, NJ). MeBr was diluted into purified air (210–240 ppm), with total flow at 10 L/min and length of exposure set at 6 or 8 h. Nose plugs were removed the following day.

### Data acquisition and analysis

Optical signals from the dorsal OB were acquired with standard wide-field epifluorescence microscopy as described previously (Bozza et al., 2004). Epifluorescence imaging was performed using an Olympus BX51 illumination turret with a 150-W Xenon arc lamp (Opti-Quip, Highland Mills, NY) at 50% intensity (attenuated with an ND50 filter), with the following fluorescence filter set from Chroma Technology (Rockingham, VT): HQ480/40 (exciter), Q505LP (dichroic), HQ535/50 (emitter), with either a 4X (0.28 N.A.) air or 20X (0.95 N.A.) water immersion objective. Odorant-evoked signals were recorded and digitized at 14-bit resolution using a back-illuminated CCD camera (NeuroCCD, SM-256; RedShirtImaging, Decatur, GA) at 256 × 256 pixel resolution and a frame rate of 7 Hz. Data acquisition was performed with Neuroplex software (RedShirtImaging).

For display in the figures, odorant-evoked response maps were spatially low-pass filtered using a Gaussian kernel (sigma values given in text) and displayed, unless where noted, with fluorescence normalized to 95% of the maximum value of that map. In order to compare maps across imaging sessions in chronically imaged animals, image registration was performed by maximizing the correlation between resting fluorescence images or, when possible, using implanted fiducial markers (see above). In acutely imaged animals, OB positions were roughly aligned using the resting fluorescence image and the midline and posterior sinus as landmarks (Wachowiak and Cohen, 2001; Bozza et al., 2004). For calculating response amplitudes and positions of input to glomeruli, regions of interest (ROIs) were defined for all spH foci in the response maps using criteria based on focus size, signal-to-noise ratio and optical signal time-course to identify presumptive activated glomeruli (Wachowiak and Cohen, 2001; Bozza et al., 2004).

Consensus response map topographies (e.g., Figure 4C) were generated as described in Wachowiak et al. (2013). Briefly, individual response maps were aligned relative to the midline and caudal sinus, normalized to their own maxima, thresholded at 50%, summed together, then smoothed with a 6 × 6 pixel mean kernel and the resulting maps renormalized and displayed as in Figure 4C. For statistical comparison of response map topographies (e.g., Figures 4D, E), maps were smoothed with a 3 × 3 pixel kernel, thresholded to include the top 70% of responses and centroids for each individual response map calculated from the mean of the positions of thresholded pixels. For comparison of centroid positions across animals, *x*- and *y*-positions were mapped to the zero point defined by the intersection of the sagittal midline and the anterior limit of the caudal sinus (Wachowiak and Cohen, 2001; Bozza et al., 2004). For determining domain separation, the sum of the *x*-position squared and the *y*-position squared (the squared displacement) was used. For the calculation of foci diameter (e.g., Figure 5), response maps were first slightly smoothed with a low-pass filter (Gaussian kernel, *σ* = 10 μm) to remove noise and odorant-evoked foci chosen for analysis based on their signal-to-noise ratio and time-course of the odorant-evoked fluorescence change. Focus sizes were measured by fitting the amplitude profiles of each ROI at perpendicular axes across each focus and taking the full-width at half-maximum (FWHM) of the fit along each axis. FWHM values for each axis were averaged to obtain a size value for each focus. To construct consensus odorant response maps for focus size analysis we projected individual, normalized response maps onto a single image using the maximal projection algorithm for confocal *z*-stacks (ImageJ).

### Two-photon imaging and analysis

All animals undergoing 2-photon laser scanning microscopy (2PLSM) at the terminal imaging session were anesthetized with pentobarbital before removal of the bone over the OB. The dura was also removed and agarose (1.2% in mouse Ringer’s) was placed onto the OB and coverslipped; petroleum jelly was used to seal the cranial window. Imaging was performed on a custom microscope that allowed for wide-field epifluorescence or multiphoton imaging through the same objective (20X, 0.95 N.A., water immersion; Olympus, Melville, NY). A 150-W Xenon arc lamp provided wide-field illumination at 2.8–6% of full intensity through the same filter set as described above. Two-photon fluorescence was excited by a mode-locked Ti:Sapphire laser (Spectra-Physics, 150 fs, 76 MHz; pumped by a 5W Millenia Vs. laser); emitted light was reflected through a mirror placed at the back aperture of the objective and directed to a bialkali photomultiplier (HC125-02, Hamamatsu Corporation, Bridgewater, NJ) fitted with an emission filter (Omega Optical, 535/45). Image acquisition was controlled by custom software in LabView (developed by Dr. J. Mertz, Boston University). For imaging odorant-evoked responses, acquisition rate was 8 Hz with a pixel resolution of 1.6 μm. Response maps obtained with 2PLSM were averaged from 5 to 10 trials to improve signal-to-noise ratio. Relative fluorescence changes were calculated using the eight frames before odorant onset as the baseline fluorescence and an average of eight frames at the peak of the evoked signal as the response. ROIs were defined using resting multiphoton resting fluorescence images.

### Confocal microscopy and histology

Following the terminal imaging session, animals were overdosed with pentobarbital and perfused intracardially with mouse Ringer’s solution (20 ml), followed by cold 4% paraformaldehyde (20 ml, 0.05 M PBS, pH 7.0). For confocal imaging, the bone surrounding the OBs was removed under alkaline PBS (0.1 M, pH 7.9) and the OBs were scanned *in situ* with a confocal microscope (LSM 510, Carl Zeiss MicroImaging Inc., Thornwood, NY) to assess OSN innervation of glomeruli using OMP-spH fluorescence. The OE and OB were then preserved in 4% paraformaldehyde until cryoprotection. Coronal sections of the olfactory tissue from the OE to OB were prepared using a cryostat at 50 μm/section. Frozen sections were counterstained with cresyl violet and mounted.

## Results

We used the gaseous olfactotoxin MeBr to unilaterally lesion the OE of mice expressing spH, an optical reporter of transmitter release, in all OSNs (OMP-spH mice; Bozza et al., 2004). Male, hemizygous OMP-spH mice were used in all experiments and lesioned at 12 weeks of age (see Section Materials and Methods). In all experiments one naris was protected from MeBr exposure with a plug that was removed after the exposure period (Cummings et al., 1997). Exposure to MeBr gas has been used extensively to lesion the OE of rats, and the severity of lesion can be controlled by varying MeBr concentration and duration of exposure (Schwob et al., 1995, 1999; Iwema et al., 2004). The time-course and cellular changes underlying degeneration and subsequent recovery of the OE after MeBr exposure have also been well-characterized (Schwob et al., 1999). Our goal was to assess the degree to which OSNs regenerate functional connections to glomeruli of the OB, where the central representations of odor information are initially formed. The general approach was to compare glomerular odor representations using spH-mediated optical signals (Bozza et al., 2004) before lesion and after a recovery period.

### Long-term, chronic imaging of sensory inputs in olfactory marker protein-synaptopHluorin (OMP-spH) mice

It was first necessary to establish the stability of odorant representations over a time-period sufficient to allow for OSN recovery—at least 60 days (Schwob et al., 1999)—and under conditions that allow for repeated optical imaging in the same animal. We have previously shown that OSN inputs can be chronically imaged in OMP-spH mice and remain stable over at least 7 days (Bozza et al., 2004). Here, we extended this time-period. We installed a chronic imaging window over the dorsal OB (see Section Materials and Methods) and imaged odorant-evoked spH response maps in three OMP-spH mice over periods of 111, 124 and 174 days.

**Figure 1:**
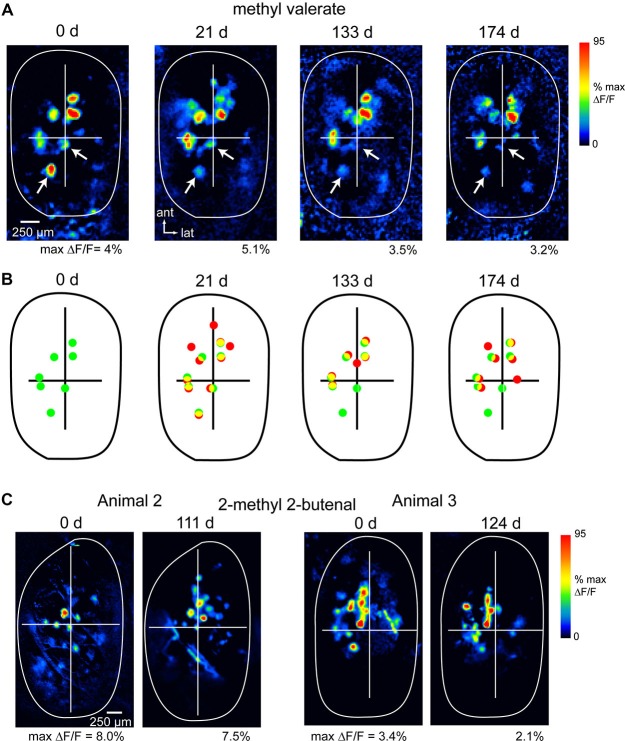
**Long-term stability of OSN representations during chronic imaging from the OB. (A)** spH response maps evoked by the same odorant (methyl valerate, 1% s.v.) imaged at four time points in the same animal (expressed as days after initial imaging session). The approximate outline of the dorsal OB is shown; crossbars are for comparisons across maps. Each map is normalized to its own maximum; absolute maximum signal amplitudes (“max ΔF/F”) are given below each map. Arrows indicate signal foci that are apparent in each map but show reduced amplitudes over time. Maps were smoothed with a Gaussian kernel (*σ* = 25 μm, kernel width = 70 μm) for display. **(B)** Comparison of spH signal foci locations for responses at day 0 and each later time-point, taken from maps in **(A)**. Dots identify signal foci with amplitudes above 30% of maximal amplitude. Green dots indicate foci in initial session, red indicates foci in the current session, yellow indicates colocalized foci. **(C)** spH response maps for two additional animals imaged at 111 and 124 days time-points; odorant: 2-methyl 2-butenal.

Figure 1 shows examples of spH response maps imaged at different time-points in three animals. In all three animals, response maps remained similar across this period. The most significant variability in maps arose from differences of up to 50% in relative signal magnitude in different glomeruli (highlighted by arrows, Figure 1A); these differences could affect the absolute number of glomeruli activated above an arbitrary threshold level. Such variability likely reflects differences in overall sensitivity in different imaging sessions, due (for example) to changes in nasal patency (Oka et al., 2009), experience-dependent plasticity (Jones et al., 2008; Kass et al., 2013) or modulatory influences (McGann et al., 2005; Pírez and Wachowiak, 2008). Nonetheless the approximate number and relative position of activated glomeruli remained consistent across imaging sessions (Figures 1B, C), indicating that the procedures involved in chronic imaging (head cap and imaging window implantation, repeated anesthesia sessions and odorant presentations) did not induce apparent changes in functional connections between OSNs and their target glomeruli.

To assess the precision with which response maps could be monitored across time-points, we compared the positions of the few (2–4) most strongly-activated glomeruli in maps evoked by the same odorant in different sessions. OB images were aligned as described in the Section Materials and Methods, then the distance between each glomerulus at the initial time-point and its nearest neighbor at the later time-point was measured. Using this measure, the average change in the position of spH signal foci between baseline and the later time-point (111–176 days) was 71.0 ± 29.1 μm (mean ± s.d*.*; *n* = 21 glomeruli taken from three animals and using nine odorants). Thus, in unlesioned animals, we are able to chronically map functional inputs to glomeruli with a spatial precision of smaller than the average diameter of a glomerulus (Bozza et al., 2004).

### MeBr exposure eliminates odorant-evoked responses in the olfactory bulb (OB)

MeBr potency has not been as extensively characterized in mice (Chen et al., 2004) as it has in rats (Schwob et al., 1995, 1999; Iwema et al., 2004); in addition, the relationship between the initial loss of OSNs and functional inputs to the OB immediately after MeBr exposure is unclear. Thus we next examined the effect of MeBr exposure on odorant-evoked spH signals and on OSN loss. In these animals spH signals were imaged shortly (4–10 days) after lesion, after which the mouse was sacrificed and damage to the OE assessed histologically. We used different MeBr exposure protocols that resulted in a range of lesion severity.

**Figure 2:**
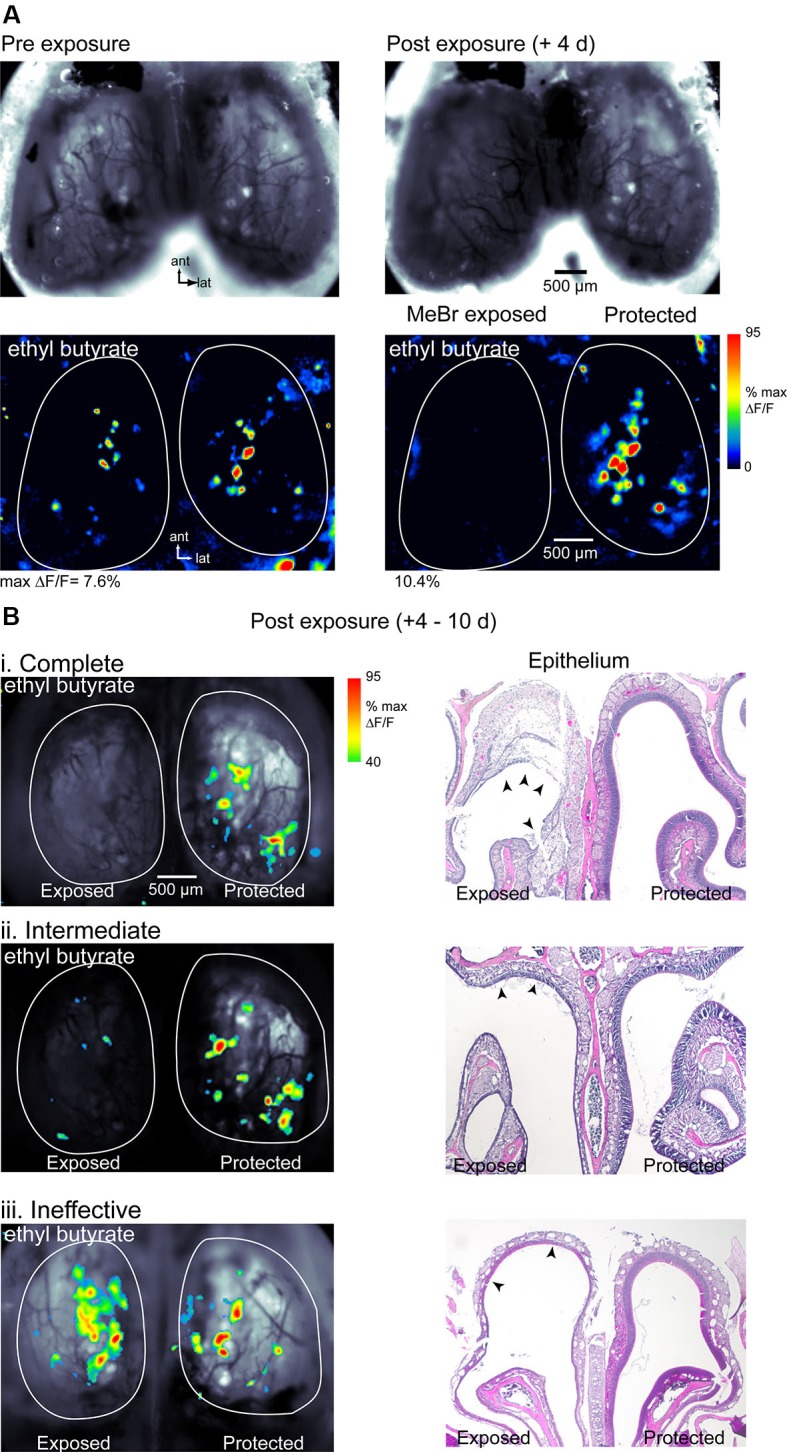
**Effect of MeBr lesion on functional OSN inputs to the OB. (A)** Resting fluorescence images (*top*) and response maps (*bottom*) imaged in the same animal 10 days before (*left*) and 4 days after (*right*) unilateral MeBr exposure. This animal showed a decrease in resting fluorescence and a complete loss of odorant-evoked spH signals on the exposed side. Responses on the protected side were unaffected. Odorant, ethyl butyrate (1% s.v.). **(B)** Response maps overlaid on resting fluorescence images (*left column*) and post-hoc H&E-stained nasal cavity sections (“Epithelium”, *right column*) for three additional animals showing different degrees of functional loss after MeBr exposure. Effects were classified as complete (**i**), intermediate (**ii**) or ineffective (**iii**) based on the amplitude and sensitivity of the odorant-evoked spH signal on the MeBr-exposed side (See the text). Response maps scaled as in **(A)** but thresholded at 40% of maximal δF/F. The nasal cavity showed widespread damage to the OE in all three animals. In the complete lesion **(i)**, the full tangential extent of the dorsomedial epithelium has been lesioned and in many areas the damage extends through the basal lamina (*arrowheads*), leading to an exudation that will become organized as endonasal scar tissue (cf. asterisks in Figure 8B). In the intermediate lesion **(ii)**, the full tangential extent of the dorsomedial epithelium is damaged but there are some residual neurons along the roof of the dorsal meatus (*arrowheads*). In the “ineffective” lesion **(iii)**, the full tangential extent of the dorsomedial epithelium is also damaged but there are residual neurons along the roof of the meatus in the area between the two *arrowheads*. Resting spH fluorescence in the OB is sharply diminished in the “complete” and “intermediate” lesions, but appears normal in the “ineffective” lesion.

At a MeBr exposure of 215 ppm for 8 h (*n* = 10 mice) or 230 ppm for 6 h (*n* = 4), exposure resulted in a complete loss of detectable odorant-evoked spH signals in half (7/14) of all mice (Figure 2A). Higher dosages resulted in significant rates of mortality (not shown). In animals showing a loss of odorant-evoked signals, resting spH fluorescence on the MeBr-exposed side was also diminished although not eliminated entirely at 4–10 days post-lesion (Figure 2A). Resting fluorescence and spH response maps remained robust on the protected side of all mice (Figure 2). In approximately 20% of MeBr-exposed animals (3 of 14), resting fluorescence and evoked spH signals were still detectable but weaker on the exposed side compared to the pre-lesion imaging session or the protected side (Figure 2B, ii). Further quantification from similarly-lesioned mice in a different cohort is provided below. In the remaining approximately 30% of animals (4 of 14), resting fluorescence and spH signals were similar in magnitude to the pre-lesion session or to those on the protected side (Figure 2B, iii). These results indicate that MeBr exposure can eliminate OSN responsiveness unilaterally and that the effectiveness of exposure is more variable than has been previously observed in rats (Schwob et al., 1995, 1999).

In this cohort, mice were sacrificed immediately after imaging and MeBr-induced damage to the OE was assessed histologically. Acutely after exposure, damaged areas were easily evident in hematoxylin and eosin (H&E)-stained sections by the sloughing of sustentacular cells and neurons, as previously described (Schwob et al., 1995). Mice showed some variation in the severity of the damage from animal to animal even when carefully controlled for age, weight and strain such that sparing was seen in some areas while in other areas damage was so severe that the basal lamina was breached leading to a serum exudate in the nasal cavity—a circumstance that precludes regeneration of the epithelium (Schwob et al., 1995). The portion of the OE that projects to the region of the dorsal OB imaged in these experiments corresponds roughly to the territory defined by lack of staining with anti-OCAM/mamFasII antibodies (Schwob and Gottlieb, 1986; Uchida et al., 2000); thus further description of the OE after lesion recovery (see below) is limited to that area.

Mice showing a complete loss of odorant-evoked spH signals were characterized by complete or near complete destruction of the neuronal and sustentacular cell populations in the dorsal half of the OE (Figure 2B, i). In these cases, the full extent of the dorsal OE was damaged based on comparison with the protected side, and across the vast majority of that epithelium the neuronal population was destroyed completely (see “exposed” side of OE image, Figure 2B, i). Mice showing weakened spH signals and classified as having intermediate functional lesion (Figure 2B, ii) also showed significant damage to the OE, but substantial areas of the dorsal epithelium were spared, particularly at posterior levels of the nasal cavity. Surprisingly, even those mice that retained robust spH signals and so were classified as having ineffective functional lesion showed at least moderate OE damage, particularly in the far anterior and far posterior nasal cavity (Figure 2B, iii). Overall, these results indicate that functional imaging of odorant-evoked spH signals is a stringent assay for the effectiveness of MeBr lesion: animals showing a complete loss of spH signal likely have only a small fraction of OSNs surviving after lesion, with larger survival rates reflected in robust odorant-evoked signals.

### Functional inputs to olfactory bulb (OB) glomeruli recover after MeBr lesion

To assess the functional recovery of OSN connections to OB glomeruli after MeBr lesion, we imaged spH odorant response maps at three time-points: 4–10 days before lesion, approximately 4 days after lesion to assess lesion effectiveness, and 12–13 weeks after lesion to assess recovery. Ten mice were unilaterally exposed to MeBr using a dosage and exposure protocol (235 ppm for 6.5 h) similar to that used to assess lesion effectiveness (above). Of these animals, four showed persistent odorant-evoked responses at the assessment session and so were excluded from further analysis; the remaining six animals showed complete loss of spH signals on the exposed side at assessment. In none of the mice did we observe any obvious behavioral changes either immediately after unilateral exposure or during the recovery period.

**Figure 3:**
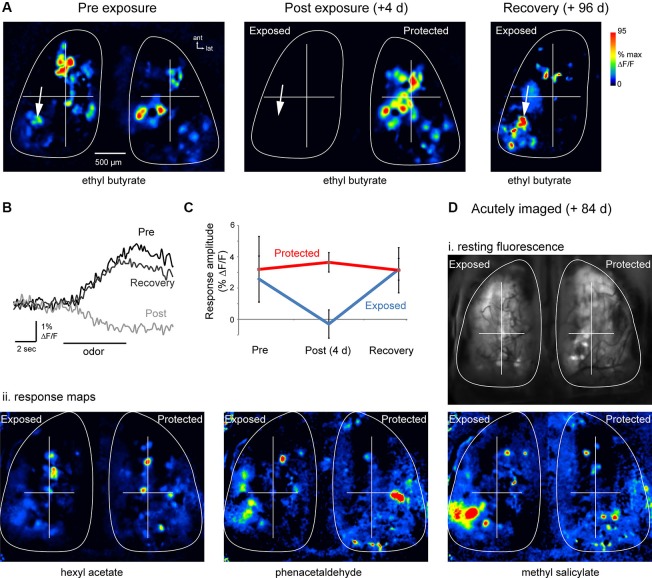
**Functional recovery of OSN inputs to the OB after MeBr lesion**. **(A)** Resting fluorescence and response maps imaged in the same animal before (“Pre-exposure”), 4 days after (“Post-exposure”) and 96 days after (“Recovery”) unilateral exposure to MeBr. Odorant was ethyl butyrate in all cases. Odorant-evoked responses were eliminated post-exposure; responses and resting fluorescence (not shown) reappeared at Recovery. **(B)** Traces showing time-course of the odorant-evoked spH signal from one location (indicated by arrow in **(A)**), which was similar before lesion and after recovery. Fluorescence decrease at the “Post” exposure time-point reflects intrinsic hemodynamic artifacts described previously. **(C)** Summary data showing spH response amplitudes on the exposed and protected sides before lesion and after recovery. See the text for details. Error bars indicate s.d. **(D)** Odorant response maps imaged in a single acute session at 84 days post unilateral MeBr exposure. **(i)** Resting spH fluorescence. **(ii)** Odorant-evoked response maps evoked by three different odorants appear similar on the exposed and protected OBs, with the most variance appearing as different relative amplitudes of the spH signal. Note presence of putative homologous individual glomeruli on each side, especially in the anterior OB. To facilitate comparison, response maps from each side were normalized separately to their own maximum (same pseudocolor scale as in **(A)**).

In all six of these mice, odorants evoked clear spH signals on the lesioned side at 12 weeks post-lesion. Figures 3A, B show odorant response maps and spH signal traces in a representative animal. Evoked spH signals 12 weeks post-lesion appeared roughly similar to those observed before lesion, consisting of spatially heterogeneous responses with numerous discrete signal foci (Figure 3A). In many cases these signal foci appeared in locations that were nearly identical to those observed before lesion (Figure 3A, arrows). Using the ROIs determined at baseline imaging for both the exposed and protected OBs, we were able to identify and measure odorant-evoked spH signals after the 12 weeks recovery period. The time-course of the odorant-evoked spH signal was also similar before lesion and after recovery (Figure 3B). Across animals, the peak amplitude of the spH signal was similar before lesion and after recovery for these animals (pre-lesion: 2.6 ± 1.5%; mean ± s.d.; recovery: 3.2 ± 0.7%; *p* = 0.45, paired *t*-test, *n* = 22 odorant pairs across six animals; Figure 3C).

To address potential confounds of the chronic imaging window on OSN recovery (Xu et al., 2007), a separate set of four mice were exposed unilaterally to MeBr (215 ppm for 8 h) and spH signals imaged in a single session 12 weeks after exposure. Thus in these mice there was no baseline session or assessment of lesion effectiveness, but odorant-evoked spH response maps were compared between the exposed and protected sides. In these animals, response maps appeared qualitatively similar to those seen on the protected OB, and included individual signal foci that were located in a position that was symmetric with foci on the unlesioned side (Figure 3D). Peak-amplitude spH signals were similar on the exposed (*n* = 28 glomeruli from four animals) and unexposed sides (*n* = 32 glomeruli, four animals; *p* = 0.35, unpaired *t*-test). Thus, OSNs that are replaced after MeBr lesion reestablish convergent functional connections to glomeruli of the OB.

### Recovery of sensory input map topography after olfactory sensory neuron (OSN) regeneration

**Figure 4:**
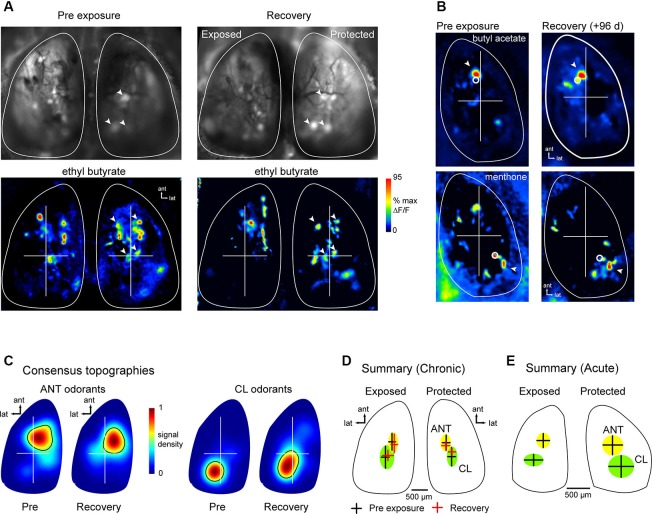
**Odor response map topography is reestablished after MeBr lesion and OSN recovery**. **(A)** spH resting fluorescence and bilateral response maps imaged before unilateral MeBr exposure (“Pre-exposure”) and after recovery in the same animal, shown for ethyl butyrate. On the protected side, blood vessel pattern and position of brightly fluorescent glomeruli is near-identical, as are locations of strongly-activated glomeruli (*arrowheads*). On the exposed side, brightly fluorescent glomeruli and spH responses are similar in amplitude and similar but not identical in location after recovery. **(B)** Additional examples showing spH response maps for two odorants that preferentially evoke input to the anterior (*top*) and caudal-lateral OB (*bottom*), respectively. For each odorant, response maps are topographically similar at both time-points. *White circles* indicate the location of the centroid of each map, calculated after smoothing and thresholding (See the text). *Arrowheads* indicates a particular spH focus (glomerulus) whose position is consistent in both pre-exposure and lesion-recovered maps. **(C)** Consensus topographies for anterior (ANT) and caudal-lateral (CL) odorant response maps compiled from chronically-imaged mice (*n* = 5 mice, 4–8 odorants per mouse) unilaterally exposed to MeBr and imaged before exposure (“Pre”) and after lesion recovery (“Recovery”). Pseudocolor scale indicates relative density of odorant-evoked spH signal across all response maps in each category. Black contour indicates arbitrary 50% cutoff of relative density plot. See the text for analysis details. **(D)** Quantitative analysis of response topographies in chronically-imaged mice (same animals and odorants as in **(C)**). Crosshairs and shaded areas show centroid locations before lesion (black crosshairs, “Pre-exposure”) and after recovery (red crosshairs, “Recovery”) for anterior (ANT, yellow) and caudal-lateral (CL, green) odorants (See the text for list). The centers of the cross hairs denote average centroid location across all pooled odorants, with the arms and ellipses extending to 1 s.d. in *x*- and *y*-directions. Domains remained distinct for each time point and similar across time-points (note that this analysis differed slightly from that used to produce consensus topographies in **(C)**; See the text for analysis details). **(E)** Centroid locations analyzed and plotted as in **(D)** for acutely-imaged animals, showing similar distribution of centroid locations for exposed and protected sides imaged in a single recovery session.

Projections of OSNs to OB glomeruli show at least two levels of spatial organization: (1) OSNs expressing a given OR converge onto a single glomerulus whose position within a domain varies by several hundred microns in different animals and remains relatively constant in the same animal over time (Strotmann et al., 2000; Schaefer et al., 2001; Costanzo and Kobayashi, 2010; see also Figure 1); and (2) projections show a broad topography in which OSNs of a particular class project within spatial domains spanning large regions of the bulbar surface (Nagao et al., 2002; Bozza et al., 2009; Pacifico et al., 2012). Chronic imaging of odorant response maps before lesion and after recovery showed that spH signals in lesion-recovered animals often differed slightly in the precise location of individual signal foci, but that spH signals remained clustered in locations similar to those seen in pre-lesion response maps (Figures 4A, B). These examples suggest that, while precise targeting of OSNs to pre-existing glomerular locations may be disrupted in regenerated OSNs, projections to the OB may recover with sufficient precision to preserve the topography of functional domains related to particular odorants.

To analyze the topography of lesion-recovered OSN projections more thoroughly, we examined response maps for odorants that have been previously shown to preferentially activate glomeruli in either the anterior (ANT) dorsal OB (aliphatic aldehydes and acids and some esters) or the caudolateral (CL) dorsal OB (ketones and aromatics) (Uchida et al., 2000; Wachowiak and Cohen, 2001; Bozza et al., 2004, 2009; Takahashi et al., 2004; Matsumoto et al., 2010). Because different odorants were tested in different animals, response maps were pooled into either ANT-activating or CL-activating groups depending on odorant identity. ANT odorants were: ethyl butyrate, hexaldehyde, 2-methyl-2-butenal, butyl acetate and butyric acid; CL odorants were: acetophenone, 2-hexanone, menthone, methyl benzoate and eugenol. For a qualitative comparison of response map topography before and after lesion recovery, we generated consensus topographies as described previously (Wachowiak et al., 2013) and in Section Materials and Methods, compiled from pre-lesion and lesion recovery imaging sessions in the same chronically-imaged animals. ANT and CL odorants evoked the strongest responses in similar OB regions before lesion and after recovery (Figure 4C).

For quantitative comparison of response map topographies, maps from ANT and CL odorants and between pre-lesion (baseline) and recovery sessions were compared using the centroid of each response map (see Section Materials and Methods; Figure 4D). Centroid positions were compared statistically using a 4-factor ANOVA with the following factors: ANT-odorants at baseline; ANT-odorants at recovery; CL-odorants at baseline and CL-odorants at recovery. There were a minimum of six response maps (at least six different odorants) for each factor; MeBr exposed and protected sides were analyzed separately. In the pre-exposure (baseline) session, as expected, maps for ANT- or CL-activating odorants were located in largely non-overlapping domains in the ANT- or CL- OB, respectively (Figure 4D), with distinct centroid positions as determined by the 4-factor ANOVA (*F*(3, 49) = 2.885, *p* < 0.05) and a post-hoc test comparing ANT- and CL-odorant centroids at baseline (Fisher’s exact test: *p* < 0.05). However, there was no significant change in ANT- or CL-odorant map topography after lesion recovery (Figure 4D), with post-hoc analyses reporting no difference in centroid locations between baseline and recovery sessions (Fisher’s exact test; ANT: *p* > 0.34; CL: *p* > 0.50). In agreement with the results in chronically-imaged animals, in the four animals that were exposed to MeBr and acutely imaged at the recovery stage, ANT- and CL-odorants evoked inputs to regions with statistically distinct centroids (unpaired Student’s *t*-test, ANT vs. CL positions, *n* = 23, *p* < 0.001), similar to those seen in the unexposed OB of the same animals (Figure 4E). These results indicate that OSNs preferentially regenerate axonal projections to targets within their original functional domains on the OB surface, thus reconstituting the broad topography of glomerular activation that is a hallmark of primary odor representations in the OB.

### Atypical convergence of olfactory sensory neurons (OSNs) to olfactory bulb (OB) targets after lesion recovery

**Figure 5:**
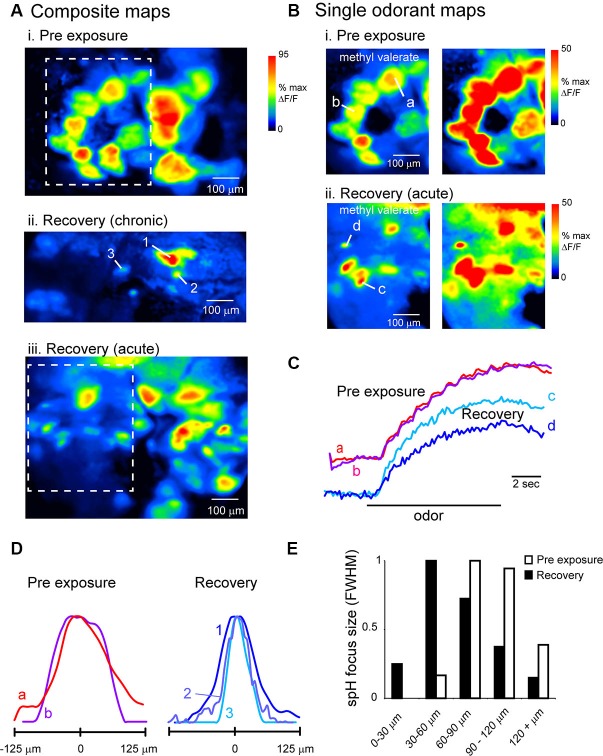
**Evidence for atypical convergence of OSNs onto OB targets after recovery from MeBr lesion**. **(A)** Composite odorant response maps imaged at higher-magnification (20X objective) in unlesioned **(i)** and lesion-recovered **(ii, iii)** OBs. Maps are maximal-value projections of responses to all odorants tested in a given session (see Section Materials and Methods). In both chronically-imaged **(ii)** and acutely-imaged **(iii)** mice, odorants evoked spH signal foci that were smaller in size than a typical glomerulus. Boxes in **(i** and **iii)** indicate regions rescaled in **(B)**. **(B)** Response maps from the regions in **(A)** evoked by a single odorant (methyl valerate, 1% s.v.), scaled to their own maximum (*left*) and to 50% of their maximum (*right*) to highlight weaker-activated regions for both Pre-exposure **(i)** and Recovery conditions **(ii)**. Smaller-sized foci are still apparent after rescaling. **(C)** Time-course of spH signal in typical and undersized foci in unlesioned (a, b) and lesion-recovered (c, d) animals. Traces taken from locations indicated in **(B)**. Unlesioned and lesion-recovered traces are offset and scaled separately to compare signal time-course. **(D)** Normalized intensity profiles through spH foci taken from response maps in unlesioned (a, b) and lesion-recovered animals (1, 2, 3; see **A**, **ii**), indicating smaller focus size in recovered animals. **(E)** Histogram of spH focus sizes for pre-exposure and lesion-recovered preparations. Lesion-recovered animals show an increase in the number of foci below 60 μm full-width at half-maximum (FWHM). Bin size of the histogram (20 μm) is 1 standard deviation of the FWHM values for pre-lesion OBs.

Close inspection of lesion-recovered response maps revealed numerous examples of spH signal foci that appeared smaller than a typical glomerulus. These smaller foci—or the presence of more diffuse spH signals—could reflect OSN axons that failed to converge or that only partially innervated a glomerulus (St. John and Key, 2003; Blanco-Hernández et al., 2012). To examine these signals more carefully we imaged responses at higher-magnification and smaller depth of field (20X, 0.95 N.A. objective, 3.5 μm pixel resolution) using the same animals as in the above analysis. Imaging at this resolution confirmed that in lesion-recovered mice, odorant-evoked spH signals often appeared in foci that were subglomerular in size (Figures 5A, B). Such foci were also apparent in acutely-imaged MeBr-treated animals (Figure 5A, iii), indicating that these were not a result of chronic window implantation. spH signals from subglomerular-sized foci displayed response dynamics that were similar to those from unexposed animals or larger foci (Figure 5C).

We quantitatively compared spH signal foci sizes in maps taken from baseline and lesion-recovered imaging sessions by fitting the signal intensity profile of discrete foci to a Gaussian and measuring the FWHM of the fit (see Section Materials and Methods and Meister and Bonhoeffer, 2001). These measurements were made in acutely-imaged, MeBr-exposed animals imaged at the recovery time-point. On the side exposed to MeBr, there was a larger number of small-diameter foci (Figures 5D, E), leading to significant reduction in the mean focus size from 90.7 ± 20.8 μm. (*n* = 45 glomeruli from three animals) to 67.6 ± 33.5 μm (*n* = 100 glomeruli from four animals; *p* < 0.0001, unpaired *t*-test). Thus, OSN inputs to lesion-recovered OBs frequently converge onto structures smaller than the size of typical glomeruli.

**Figure 6:**
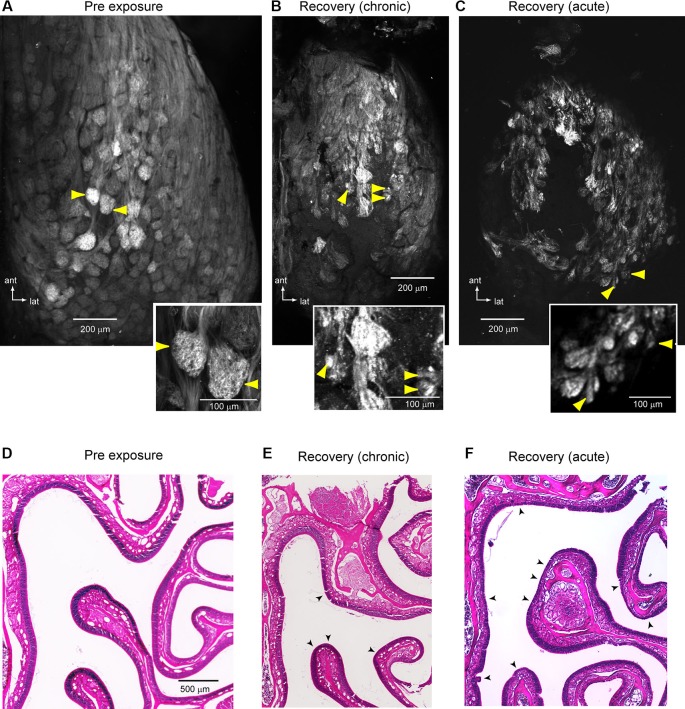
**Anatomical evidence for atypical convergence of OSNs onto OB glomeruli**. **(A)** Confocal scan of a representative unlesioned OB (maximal *z*-stack projection). Inset shows detail of the glomeruli noted by *yellow arrows*. Glomeruli are clearly delineated and relatively uniform in size. **(B, C)** Confocal scans from two lesion-recovered animals, one chronically-imaged **(B)** and one acutely imaged **(C)**. In both cases, glomerular borders are less distinct and OSNs often terminate in smaller structures (*yellow arrowheads, detail in insets*). In addition, regions of the central dorsal OB appear to lack innervation by OSNs. **(D–F)** H&E-stained sections of the OE from the same animals shown in **(A–C)**. In **(E)**, there is substantial but not complete recovery of the epithelium. *Arrowheads* indicate areas with a reduced contingent of neurons as compared to the protected side. In **(F)**, much of the epithelium remains less than fully recovered. *Arrowheads* indicate areas that are grossly abnormal and completely lacking in neurons.

To investigate the underlying anatomical structure of lesion-recovered OSN inputs to the OB, we used confocal microscopy to scan the intact dorsal OB of imaged preparations (see Section Materials and Methods). In control animals and on the protected side of unilaterally-lesioned animals, OSN axons formed large bundles that coalesced into well-defined glomeruli defined by discrete, roughly spherical areas of OSN axon terminals (Figure 6A). In contrast, in lesion-recovered OBs OSNs often converged onto smaller structures and glomerular boundaries appeared less well-defined (Figures 6B, C). Qualitatively similar results were seen in chronically- and acutely-imaged lesion-recovered animals. Finally, nearly all lesion-recovered OBs showed at least some regions of the dorsal OB with no clear spH fluorescence, indicating a lack of reinnervation by OSNs (Figures 6B, C). The OE of these preparations had undergone substantial, although incomplete, reconstitution of the OSN population (Figures 6D–F). For example, the chronically-imaged mouse shown in Figures 6B, E showed substantial regeneration of the OE but nonetheless had patches where the OE thickness was thinner than the contralateral, unlesioned side (Figure 6E). Similarly, in the acutely-imaged example shown in Figures 6C, F, a greater extent of the dorsal OE does not recover fully or at all (Figure 6F), consistent with the lesser degree of glomerular reinnervation that was observed in this animals (Figure 6C).

**Figure 7:**
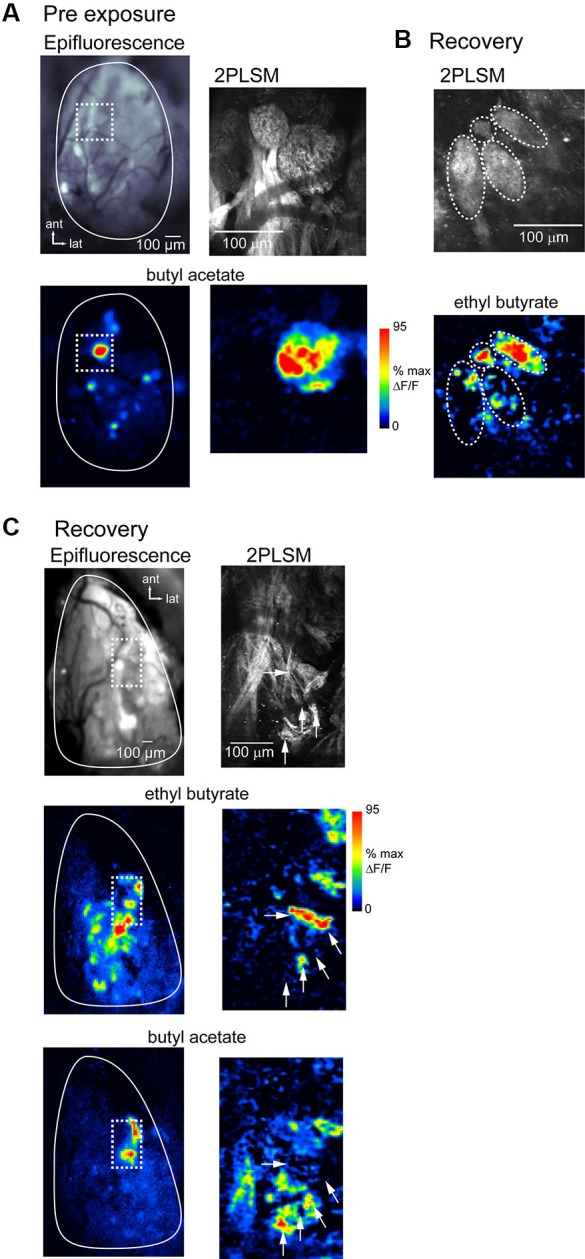
**Atypical glomerular convergence of OSNs confirmed with *in vivo* two-photon imaging of spH signals.** **(A)** Resting fluorescence and evoked spH response maps imaged with wide-field epifluorescence (*left*) and 2-photon laser scanning microscopy (2PLSM; *right*) in the same unlesioned animal. Epifluorescence image taken with 4x objective. Odorant, butyl acetate. Dashed box indicates area imaged at higher-magnification with 2PLSM. With 2PLSM, spH fluorescence increases are apparent throughout the glomerulus, with “hot-spots” of high signal amplitude in subglomerular regions. **(B)** 2PLSM resting fluorescence (*top*) and response map (*bottom*) from a lesion-recovered animal. Glomerular boundaries are less well-defined (compare to **(A)**); in this example several relatively discrete structures are apparent (indicated by dashed ovals), one of which is only 30–40 μm in diameter (*top*). Response map (*bottom*) shows spH signals with approximate boundaries of the four structures overlaid. In two of these structures, odorant (ethyl butyrate, 5% s.v.) only evokes signals in a few foci, with the rest of the area showing no response. **(C)** Low-magnification wide-field (*left*) and high-magnification 2PLSM imaging (*right*) from another lesion-recovered animal, showing odorant-specific distribution of spH signals lacking a clear glomerular structure.*Top*: Resting fluorescence of the imaged regions. 2PLSM image is a projection of a *z*-stack through the olfactory nerve and glomerular layers. *Middle, bottom*: response maps evoked by ethyl butyrate and butyl acetate. Epifluorescence maps are unsmoothed (unlike previous figures). spH signals imaged with 2PLSM** from the regions containing the strongest responses to both odorants (dashed box) reveal no clear glomerular structure from resting fluorescence. Instead, signals are distributed in small “hot-spots” (*white arrows*) whose distribution differs for the two odorants.

To directly compare spH signal foci with the underlying anatomical structure of OSN inputs in lesion-recovered animals, we imaged spH signals using *in vivo* 2PLSM in a subset of preparations (*n* = 3 chronically-imaged, 3 acutely-imaged, and 2 unexposed animals). Figure 7A shows resting fluorescence and odorant-evoked response maps imaged with wide-field epifluorescence and with 2PLSM in an unlesioned animal. OSN innervation of distinct glomeruli is clearly resolved *in vivo* using 2PLSM, and odorant-evoked spH signals are readily detectable. Odorants evoke spH signals throughout the glomerulus but with hot spots in smaller domains within it (Figure 6A), in agreement with previous reports (Wachowiak et al., 2004).

spH signals imaged with 2PLSM in lesion-recovered animals revealed qualitatively different signals with respect to glomerular structure. OSN axons often converged to atypically small structures (Figure 7B) or failed to delineate glomeruli with clear boundaries (Figure 7C). In these areas odorants often evoked spH signals appearing as “hot-spots” that appeared in only a portion of the glomerular structure (Figure 7B). Nonetheless, different odorants evoked different spatial distributions of spH signals (Figure 7C), consistent with their activating distinct (although smaller) populations of convergent OSNs. Overall, these results suggest that the smaller-sized spH signal foci observed in lesion-recovered OBs reflect OSN axon projections that do not converge to a canonical glomerular structure but which nonetheless provide functional input to OB targets.

### Functional recovery of olfactory sensory neuron (OSN) inputs after severe and lasting damage to the OE

Exposure to higher doses of MeBr can lead to more pronounced damage to the OE that allows for only a limited recovery and regeneration of OSNs (Schwob et al., 1995, 1999). To test the limits at which OSNs can recover functional connections to the OB, we unilaterally exposed an additional six animals to a higher MeBr dosage (240 ppm, 8 h). This dosage was lethal in all but three animals. In these animals, spH signals were imaged in a single, acute session after the 12 week recovery period.

**Figure 8:**
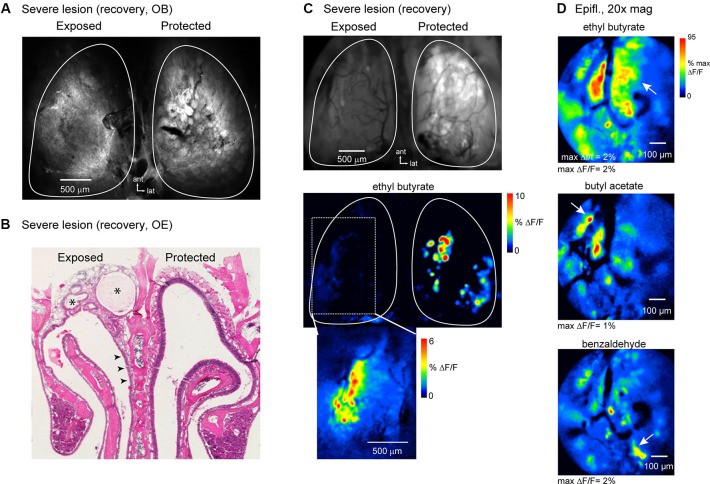
**OSNs can partially reestablish functional inputs to the OB after severe and lasting trauma to the olfactory epithelium**. **(A)** Confocal scan (maximal projection) of the dorsal OBs of an animal imaged at recovery stage after high-dosage unilateral MeBr exposure (See the text). The OB on the protected side appears normal; the OB on the exposed side fails to show OSNs terminating in glomerular structures. **(B)** H&E-stained section of OE from the animal shown in **(A)**. On the lesioned side there is almost no reconstitution of the neuronal population, although neurons are apparent in some areas of the epithelium (*arrowheads*). Scar tissue fills much of the dorsal meatus in this animal (asterisks). **(C)** Epifluorescence image and spH response map from the same animal as in **(A, B)** imaged at the recovery stage. Resting fluorescence is low on the exposed side. Very weak odorant-evoked spH signals were detected on this side and were confined to the lateral margin of the dorsal OB (*bottom*). Inset shows evoked signals from this region after rotating the head for improved optical access and scaling responses in this region to their own maximum. **(D)** Response maps evoked by ethyl butyrate and two additional odorants imaged from the same animal at higher-magnification (20X objective) with wide-field optics. Multiple spH signal foci, nearly all of which are subglomerular in size, are evoked by each odorant, although the patterns of activation are distinct. Arrows indicate signal foci that appear specific for a given odorant.

Confocal scans of the dorsal OB of these animals showed reduced resting spH fluorescence and no clear glomerular structure (Figure 8A); the OB on the protected side appeared normal. Histological examination of the OE of these animals showed extensive and lasting damage on the exposed side, such that there was little reconstitution of the neuronal population (Figure 8B). Instead, the majority of the epithelium had undergone respiratory metaplasia, in which damaged OE is replaced by respiratory epithelium after destruction of globose basal cells by severe MeBr exposure (Schwob et al., 1995; Jang et al., 2003). In these cases, the architecture of the epithelium and underlying lamina propria is distorted by fibrosis within what was the nasal cavity and the formation of synechiae bridging across the cavity from turbinate to septum (Figure 8B). In all three mice, this type of scarring was more prevalent in the anterior nasal cavity.

*In vivo*, the OB on the lesioned side of all three animals showed greatly reduced resting spH fluorescence (mean ± s.d., lesion: 4153 ± 516 arbitrary fluorescence units, unexposed: 8884 ± 981, *n* = 3 animals), indicative of poor regeneration of OSN inputs (Figure 8C). In addition, odorant-evoked spH signals were severely attenuated. However, in each of the animals at least some odorants evoked weak but detectable spH signals; in all cases these were confined to the lateral margins of the dorsal OB (Figures 8C, D). Higher-magnification (20x) imaging of this region revealed numerous small spH signal foci or diffuse, nonfocal signals. Despite the small amplitudes and greatly perturbed spatial organization of spH signals in these animals, different odorants still evoked spatially distinct response patterns (Figure 8D). These results indicate that at least some OSNs are capable of regenerating and reestablishing odorant-specific functional connections with the OB even in the face of severe and lasting damage to the OE.

## Discussion

We assessed the capacity of the mammalian olfactory system to reestablish functional connections to the CNS and to recapitulate odor representations at the level of the OB after wholesale destruction of the OSN population. By imaging odorant-evoked spH signals from OSNs to OB glomeruli before peripheral lesion and after a 12 week recovery period, we found that this regenerative capacity is robust: odor “maps” involving many glomeruli (and thus many ORs) were reconstituted with little or no change in their topographic organization across the dorsal OB. We also obtained evidence that mistargeted OSNs—which have previously only been observed anatomically—make functional connections to the OB. Finally, we found that OSNs were able to at least partially reestablish functional connections to the OB even after lesions severe enough to permit only minor recovery of the OSN population. These results expand on earlier anatomical studies that have reported regeneration and glomerular convergence of a few OR- and histologically-defined OSN populations (Schwob et al., 1999; Costanzo, 2000; St. John and Key, 2003; McMillan Carr et al., 2004; Blanco-Hernández et al., 2012) and are consistent with a recent report that discriminative odor memories are preserved after OSN lesion and recovery (Blanco-Hernández et al., 2012).

Several lines of evidence suggest that the process of installing a chronic imaging window did not substantially affect OSN targeting. First, in unlesioned (but windowed) controls, we found that odor maps remained stable for at least 13 and for as long as 25 weeks. Second, in unilaterally-lesioned animals, we observed differences in the fine structure of response maps (described below) between the MeBr-exposed and protected sides, despite the presence of an imaging window on each side. Third, we obtained qualitatively and quantitatively similar results in animals imaged only at the 12 week recovery time-point and exposed bilaterally to MeBr. Thus, it is unlikely that the chronic imaging procedure or unilateral lesion affected the process of OSN regeneration and targeting to glomeruli.

### Recovery of odor map topography after lesion

In the mammalian OB, many odorants preferentially evoke activity in glomeruli clustered in spatial domains covering several hundred microns (Imamura et al., 1992; Uchida et al., 2000; Johnson et al., 2002). These domains are associated with odorants of a particular chemical class and are innervated by molecularly and functionally distinct OSN types (Bozza et al., 2004, 2009; Takahashi et al., 2004; Matsumoto et al., 2010; Pacifico et al., 2012). We found that this domain organization, as assessed functionally across the dorsal OB, is largely preserved after OE regeneration. This result does not simply reflect the reconstitution of normal zones of odorant receptor (OR) expression in the OE and the maintenance of rhinotopic projections from the OE to OB (Schoenfeld et al., 1994; Cummings et al., 2000; Iwema et al., 2004), as OSNs projecting to OB domains are interspersed in the OE (Bozza et al., 2009). The reconstitution of functional topography after OSN regeneration is consistent with earlier anatomical studies examining the targeting of P2- or M72-expressing OSNs or of immunohistochemically-defined OSN subsets (Cummings et al., 2000; St. John and Key, 2003; McMillan Carr et al., 2004; Blanco-Hernández et al., 2012); these studies found that OSNs project to glomeruli in topographically similar locations as in control animals, although with clear errors in targeting. The fact that, in this study, spH response maps—even those involving many glomeruli—retain a spatial organization that matches that before lesion suggests that regenerated OSN axons do not randomly converge onto OB glomeruli but instead preferentially target their appropriate domain on the OB surface. In addition we note that many lesion-recovered response maps included individual signal foci (i.e., glomeruli) that appeared in a similar location to that observed before lesion (e.g., Figure 4A) or to that of a focus on the unexposed side (e.g., Figure 3D), suggesting that the precision of targeting in regenerated OSNs may be higher than of a functional domain.

The mechanisms mediating this targeting remain unclear but may include OR identity (Feinstein et al., 2004), OSN cell type (Bozza et al., 2009) and axon guidance cues (Schwob, 2002; Schwarting and Henion, 2011). In many systems such guidance mechanisms function only during a restricted developmental window; our results suggest that they are at least partially effective in guiding OSN axons in adults. The precise targeting of OSNs to glomeruli may also be affected by activity-dependent mechanisms driven by exposure to ambient odorants (Nakatani et al., 2003; Zou et al., 2004; Kerr and Belluscio, 2006). Examining the recovery of OSNs expressing markers for ORs for which ligands are known in combination with functional imaging of inputs to all glomeruli will be useful for testing this possibility.

It remains possible that a minority of OSNs expressing the same OR fail to converge onto glomeruli in their appropriate domains, or that a minority of OSN populations converge onto glomeruli in inappropriate locations. Quantifying the degree to which such mistargeting occurs is difficult since functional domains can only be loosely defined by odorant responsiveness (Wachowiak and Cohen, 2001; Bozza et al., 2004; Wachowiak et al., 2013) (i.e., nearly all odorants activate at least some OSN input to glomeruli outside of their preferred domain) and because molecular (e.g., OR-based) tags to define domain boundaries are themselves derived from OSN convergence patterns (Bozza et al., 2009; Pacifico et al., 2012). It is also possible that spH signal foci that occur in similar locations before and after lesion may reflect activation of OSNs that express different ORs but have a similar odorant-specificity. An ideal approach to more precisely define the precision of OSN retargeting would be to tag the postsynaptic neurons associated with a given OSN population—for example, using transysnaptic transgene expression driven by OSNs expressing a particular OR.

### olfactory sensory neuron (OSN) convergence onto individual glomeruli

Another fundamental feature of OSN projections to the OB is the exclusive convergence of OSNs expressing the same OR onto a glomerulus (Mombaerts et al., 1996; Treloar et al., 2002). We found evidence for errors in this convergence after OE regeneration: lesion-recovered animals showed an increase in the number of small-sized (<60 μm) spH foci compared to controls, indicating either reduced numbers of OSN axons forming a glomerulus or partial innervation of a glomerulus by OSNs expressing a given OR. We found evidence for both possibilities using high-resolution two-photon imaging of spH signals in register with the underlying structure of OSN axons. The time-course and odorant-specificity of spH signals in these subglomerular foci was similar to spH signals in larger foci or in unlesioned controls, suggesting that transmitter release from these OSN terminals was functional. These results suggest that regenerated OSNs can provide functional input to mistargeted glomeruli and that this mistargeting is a general phenomenon seen across many OR types.

OSN mistargeting may impact odor perception after OE lesion and recovery (Yee and Costanzo, 1998; Vedin et al., 2004). In humans, dysosmias are often reported after trauma to the OE (Doty, 1979; Meisami et al., 1998). Innervation of a single glomerulus by multiple OSN types might underlie these effects. Given our evidence that OSN mistargeting is restricted to within a functional OB domain, one prediction is that discrimination of structurally similar odorants will be impaired after OE lesion and recovery, while a second is that discrimination between odorants activating different domains will be unaffected. The former prediction has not, to our knowledge, been tested. The latter prediction is supported by a recent report that behavioral discriminations of two odorants that activate distinct OB domains persist after OSN lesion and recovery (Blanco-Hernández et al., 2012). However, a second study found that odor discriminations are impaired even after partial lesion that spares many OSNs (Bracey et al., 2013), in apparent contradiction to the recovery of odor memories after lesion and regeneration. Thus, rigorously testing perceptual effects of OSN mistargeting may be difficult and will likely require combining multiple perceptual assays with imaging of odor maps in the same animals (Bracey et al., 2013).

### Factors affecting olfactory sensory neuron (OSN) regeneration and axon targeting

While we evaluated the recovery of sensory inputs to the OB after MeBr-induced OSN lesion, previous studies have used a variety of lesion models with qualitatively distinct results. Severing the olfactory nerve at the cribriform plate, which leads to extensive OSN death and subsequent regeneration, results in more extensive mistargeting after regeneration—including a loss of rhinotopic projections—than does chemically lesioning OSNs (Costanzo, 2000; Christensen et al., 2001). There also appears to be a correlation between the numbers of OSNs surviving the lesion and the degree of mistargeting (Schwob et al., 1999). For example, retargeting of P2-expressing OSNs to their appropriate glomerulus is normal if these neurons alone are selectively lesioned using a genetic method while all other OSNs are spared (Gogos et al., 2000), and chemical lesions that spare the lamina propria appear to permit more precise targeting of recovered OSNs (Blanco-Hernández et al., 2012).

After severe OE lesion with higher MeBr doses in which there was substantial lasting damage to the OE, we found that odor response maps were severely disrupted, with little or no regeneration of OSN inputs to the dorsal OB and a lack of convergence onto clear glomeruli in the lateral OB. Despite this severe disruption, however, odorants did evoke spH signals in reinnervated areas and did so with odorant-specific (though poorly-defined) spatial patterns, indicating that OSNs are capable of establishing functional inputs to the OB even in the absence of glomerular convergence. Thus the capacity of the OE to reestablish connections to the CNS appears to persist even in the face of extreme peripheral damage.

## Author Contributions

Man C. Cheung, Matt Wachowiak and James E. Schwob designed the experiments, Man C. Cheung performed the imaging experiments and data analysis, James E. Schwob administered the MeBr lesions, James E. Schwob and Woochan Jang performed the histological analysis and Matt Wachowiak and Man C. Cheung wrote the paper.

## Conflict of interest statement

The authors declare that the research was conducted in the absence of any commercial or financial relationships that could be construed as a potential conflict of interest.

## References

[B1] Blanco-Hernández E. Valle-Leija P. Zomosa-Signoret V. Drucker-Colín R. Vidaltamayo R. (2012). Odor memory stability after reinnervation of the olfactory bulb. PLoS One 7:e46338 10.1371/journal.pone.0046338 23071557 PMC3468571

[B2] Bozza T. McGann J. P. Mombaerts P. Wachowiak M. (2004). In vivo imaging of neuronal activity by targeted expression of a genetically encoded probe in the mouse. Neuron 42, 9–21 10.1016/s0896-6273(04)00144-8 15066261

[B3] Bozza T. Vassalli A. Fuss S. Zhang J. J. Weiland B. Pacifico R. (2009). Mapping of class I and class II odorant receptors to glomerular domains by two distinct types of olfactory sensory neurons in the mouse. Neuron 61, 220–233 10.1016/j.neuron.2008.11.010 19186165 PMC3013286

[B4] Bracey E. F. Pichler B. Schaefer A. T. Wallace D. J. Margrie T. W. (2013). Perceptual judgements and chronic imaging of altered odour maps indicate comprehensive stimulus template matching in olfaction. Nat. Commun. 4:2100 10.1038/ncomms3100 23820818 PMC3715885

[B5] Chen X. Fang H. Schwob J. E. (2004). Multipotency of purified, transplanted globose basal cells in olfactory epithelium. J. Comp. Neurol. 469, 457–474 10.1002/cne.11031 14755529

[B6] Christensen M. D. Holbrook E. H. Costanzo R. M. Schwob J. E. (2001). Rhinotopy is disrupted during the re-innervation of the olfactory bulb that follows transection of the olfactory nerve. Chem. Senses 26, 359–369 10.1093/chemse/26.4.359 11369671

[B7] Costanzo R. M. (2000). Rewiring the olfactory bulb: changes in odor maps following recovery from nerve transection. Chem. Senses 25, 199–205 10.1093/chemse/25.2.199 10781027

[B8] Costanzo R. M. Kobayashi M. (2010). Age-related changes in P2 odorant receptor mapping in the olfactory bulb. Chem. Senses 35, 417–426 10.1093/chemse/bjq029 20231263 PMC2871779

[B9] Cummings D. M. Emge D. K. Small S. L. Margolis F. L. (2000). Pattern of olfactory bulb innervation returns after recovery from reversible peripheral deafferentation. J. Comp. Neurol. 421, 362–373 10.1002/(sici)1096-9861(20000605)421:3<362::aid-cne5>3.0.co;2-8 10813792

[B10] Cummings D. M. Henning H. E. Brunjes P. C. (1997). Olfactory bulb recovery after early sensory deprivation. J. Neurosci. 17, 7433–7440 9295389 10.1523/JNEUROSCI.17-19-07433.1997 PMC6573448

[B11] Doty R. L. (1979). A review of olfactory dysfunctions in man. Am. J. Otolaryngol. 1, 57–79 10.1016/s0196-0709(79)80010-1 399716

[B12] Feinstein P. Bozza T. Rodriguez I. Vassalli A. Mombaerts P. (2004). Axon guidance of mouse olfactory sensory neurons by odorant receptors and the beta2 adrenergic receptor. Cell 117, 833–846 10.1016/j.cell.2004.05.013 15186782

[B13] Gogos J. A. Osborne J. Nemes A. Mendelsohn M. Axel R. (2000). Genetic ablation and restoration of the olfactory topographic map. Cell 103, 609–620 10.1016/s0092-8674(00)00164-1 11106731

[B14] Imamura K. Mataga N. Mori K. (1992). Coding of odor molecules by mitral/tufted cells in rabbit olfactory bulb. I. Aliphatic compounds. J. Neurophysiol. 68, 1986–2002 1491253 10.1152/jn.1992.68.6.1986

[B15] Iwema C. L. Fang H. Kurtz D. B. Youngentob S. L. Schwob J. E. (2004). Odorant receptor expression patterns are restored in lesion-recovered rat olfactory epithelium. J. Neurosci. 24, 356–369 10.1523/jneurosci.1219-03.2004 14724234 PMC6729985

[B16] Jang W. Youngentob S. L. Schwob J. E. (2003). Globose basal cells are required for reconstitution of olfactory epithelium after methyl bromide lesion. J. Comp. Neurol. 460, 123–140 10.1002/cne.10642 12687701 PMC3871194

[B17] Johnson B. A. Ho S. L. Xu Z. Yihan J. S. Yip S. Hingco E. E. (2002). Functional mapping of the rat olfactory bulb using diverse odorants reveals modular responses to functional groups and hydrocarbon structural features. J. Comp. Neurol. 449, 180–194 10.1002/cne.10284 12115688

[B18] Jones S. V. Choi D. C. Davis M. Ressler K. J. (2008). Learning-dependent structural plasticity in the adult olfactory pathway. J. Neurosci. 28, 13106–13111 10.1523/jneurosci.4465-08.2008 19052201 PMC2613972

[B19] Kass M. D. Moberly A. H. Rosenthal M. C. Guang S. A. McGann J. P. (2013). Odor-specific, olfactory marker protein-mediated sparsening of primary olfactory input to the brain after odor exposure. J. Neurosci. 33, 6594–6602 10.1523/jneurosci.1442-12.2013 23575856 PMC3865540

[B20] Kerr M. A. Belluscio L. (2006). Olfactory experience accelerates glomerular refinement in the mammalian olfactory bulb. Nat. Neurosci. 9, 484–486 10.1038/nn1673 16547509

[B21] Lam Y.-W. Cohen L. B. Wachowiak M. Zochowski M. R. (2000). Odors elicit three different oscillations in the turtle olfactory bulb. J. Neurosci. 20, 749–762 10632604 10.1523/JNEUROSCI.20-02-00749.2000 PMC6772422

[B22] Matsumoto H. Kobayakawa K. Kobayakawa R. Tashiro T. Mori K. Sakano H. (2010). Spatial arrangement of glomerular molecular-feature clusters in the odorant-receptor class domains of the mouse olfactory bulb. J. Neurophysiol. 103, 3490–3500 10.1152/jn.00035.2010 20393058

[B23] McGann J. P. Pírez N. Gainey M. A. Muratore C. Elias A. S. Wachowiak M. (2005). Odorant representations are modulated by intra- but not interglomerular presynaptic inhibition of olfactory sensory neurons. Neuron 48, 1039–1053 10.1016/j.neuron.2005.10.031 16364906

[B24] McMillan Carr V. Ring G. Youngentob S. L. Schwob J. E. Farbman A. I. (2004). Altered epithelial density and expansion of bulbar projections of a discrete HSP70 immunoreactive subpopulation of rat olfactory receptor neurons in reconstituting olfactory epithelium following exposure to methyl bromide. J. Comp. Neurol. 469, 475–493 10.1002/cne.11020 14755530

[B25] Meisami E. Mikhail L. Baim D. Bhatnagar K. P. (1998). Human olfactory bulb: aging of glomeruli and mitral cells and a search for the accessory olfactory bulb. Ann. N Y Acad. Sci. 855, 708–715 10.1111/j.1749-6632.1998.tb10649.x 9929675

[B26] Meister M. Bonhoeffer T. (2001). Tuning and topography in an odor map on the rat olfactory bulb. J. Neurosci. 21, 1351–1360 11160406 10.1523/JNEUROSCI.21-04-01351.2001 PMC6762249

[B27] Mombaerts P. Wang F. Dulac C. Chao S. K. Nemes A. Mendelsohn M. (1996). Visualizing an olfactory sensory map. Cell 87, 675–686 10.1016/s0092-8674(00)81387-2 8929536

[B28] Nagao H. Yamaguchi M. Takahash Y. Mori K. (2002). Grouping and representation of odorant receptors in domains of the olfactory bulb sensory map. Microsc. Res. Tech. 58, 168–175 10.1002/jemt.10146 12203695

[B29] Nakatani H. Serizawa S. Nakajima M. Imai T. Sakano H. (2003). Developmental elimination of ectopic projection sites for the transgenic OR gene that has lost zone specificity in the olfactory epithelium. Eur. J. Neurosci. 18, 2425–2432 10.1046/j.1460-9568.2003.02998.x 14622143

[B30] Oka Y. Takai Y. Touhara K. (2009). Nasal airflow rate affects the sensitivity and pattern of glomerular odorant responses in the mouse olfactory bulb. J. Neurosci. 29, 12070–12078 10.1523/jneurosci.1415-09.2009 19793965 PMC6666155

[B31] Pacifico R. Dewan A. Cawley D. Guo C. Bozza T. (2012). An olfactory subsystem that mediates high-sensitivity detection of volatile amines. Cell Rep. 2, 76–88 10.1016/j.celrep.2012.06.006 22840399 PMC3408605

[B32] Pírez N. Wachowiak M. (2008). In vivo modulation of sensory input to the olfactory bulb by tonic and activity-dependent presynaptic inhibition of receptor neurons. J. Neurosci. 28, 6360–6371 10.1523/jneurosci.0793-08.2008 18562606 PMC2566846

[B33] Schaefer M. L. Finger T. E. Restrepo D. (2001). Variability of position of the P2 glomerulus within a map of the mouse olfactory bulb. J. Comp. Neurol. 436, 351–362 10.1002/cne.1072 11438935

[B34] Schoenfeld T. A. Clancy A. N. Forbes W. B. Macrides F. (1994). The spatial organization of the peripheral olfactory system of the hamster. part I: receptor neuron projections to the main olfactory bulb. Brain Res. Bull. 34, 183–210 10.1016/0361-9230(94)90059-0 8055347

[B35] Schwarting G. A. Henion T. R. (2011). Regulation and function of axon guidance and adhesion molecules during olfactory map formation. J. Cell. Biochem. 112, 2663–2671 10.1002/jcb.23203 21618591 PMC3376016

[B36] Schwob J. E. (2002). Neural regeneration and the peripheral olfactory system. Anat. Rec. 269, 33–49 10.1002/ar.10047 11891623

[B37] Schwob J. E. Gottlieb D. I. (1986). The primary olfactory projection has two chemically distinct zones. J. Neurosci. 6, 3393–3404 3772438 10.1523/JNEUROSCI.06-11-03393.1986 PMC6568485

[B38] Schwob J. E. Youngentob S. L. Mezza R. C. (1995). Reconstitution of the rat olfactory epithelium after methyl bromide-induced lesion. J. Comp. Neurol. 359, 15–37 10.1002/cne.903590103 8557844

[B39] Schwob J. E. Youngentob S. L. Ring G. Iwema C. L. Mezza R. C. (1999). Reinnervation of the rat olfactory bulb after methyl bromide-induced lesion: timing and extent of reinnervation. J. Comp. Neurol. 412, 439–457 10.1002/(sici)1096-9861(19990927)412:3<439::aid-cne5>3.0.co;2-h 10441232

[B40] St. John J. Key B. (2003). Axon mis-targeting in the olfactory bulb during regeneration of olfactory neuroepithelium. Chem. Senses 28, 773–779 10.1093/chemse/bjg068 14654445

[B41] Strotmann J. Conzelmann S. Beck A. Feinstein P. Breer H. Mombaerts P. (2000). Local permutations in the glomerular array of the mouse olfactory bulb. J. Neurosci. 20, 6927–6938 10995837 10.1523/JNEUROSCI.20-18-06927.2000 PMC6772838

[B42] Takahashi Y. K. Kurosaki M. Hirono S. Mori K. (2004). Topographic representation of odorant molecular features in the rat olfactory bulb. J. Neurophysiol. 92, 2413–2427 10.1152/jn.00236.2004 15152015

[B43] Treloar H. B. Feinstein P. Mombaerts P. Greer C. A. (2002). Specificity of glomerular targeting by olfactory sensory axons. J. Neurosci. 22, 2469–2477 11923411 10.1523/JNEUROSCI.22-07-02469.2002 PMC6758332

[B44] Uchida N. Takahashi Y. K. Tanifuji M. Mori K. (2000). Odor maps in the mammalian olfactory bulb: domain organization and odorant structural features. Nat. Neurosci. 3, 1035–1043 10.1038/79857 11017177

[B45] Vedin V. Slotnick B. Berghard A. (2004). Zonal ablation of the olfactory sensory neuroepithelium of the mouse: effects on odorant detection. Eur. J. Neurosci. 20, 1858–1864 10.1111/j.1460-9568.2004.03634.x 15380007

[B46] Wachowiak M. Cohen L. B. (2001). Representation of odorants by receptor neuron input to the mouse olfactory bulb. Neuron 32, 723–735 10.1016/s0896-6273(01)00506-2 11719211

[B47] Wachowiak M. Denk W. Friedrich R. W. (2004). Functional organization of sensory input to the olfactory bulb glomerulus analyzed by two-photon calcium imaging. Proc. Natl. Acad. Sci. U S A 101, 9097–9102 10.1073/pnas.0400438101 15184670 PMC428479

[B48] Wachowiak M. Economo M. N. Díaz-Quesada M. Brunert D. Wesson D. W. White J. A. (2013). Optical dissection of odor information processing in vivo using gcamps expressed in specified cell types of the olfactory bulb. J. Neurosci. 33, 5285–5300 10.1523/jneurosci.4824-12.2013 23516293 PMC3690468

[B49] Xu H. T. Pan F. Yang G. Gan W. B. (2007). Choice of cranial window type for in vivo imaging affects dendritic spine turnover in the cortex. Nat. Neurosci. 10, 549–551 10.1038/nn1883 17417634

[B50] Yee K. K. Costanzo R. M. (1998). Changes in odor quality discrimination following recovery from olfactory nerve transection. Chem. Senses 23, 513–519 10.1093/chemse/23.5.513 9805635

[B51] Zou D.-J. Feinstein P. Rivers A. L. Mathews G. A. Kim A. Greer C. A. (2004). Postnatal refinement of peripheral olfactory projections. Science 304, 1976–1979 10.1126/science.1093468 15178749

